# 3-(Methoxycarbonylmethylene)isobenzofuran-1-imines as a New Class of Potential Herbicides

**DOI:** 10.3390/molecules19068261

**Published:** 2014-06-18

**Authors:** Fabrizio Araniti, Raffaella Mancuso, Ida Ziccarelli, Francesco Sunseri, Maria Rosa Abenavoli, Bartolo Gabriele

**Affiliations:** 1 Dipartimento AGRARIA, Università Mediterranea di Reggio Calabria, Reggio Calabria 89124, Italy; 2 Dipartimento di Chimica e Tecnologie Chimiche, Università della Calabria, Via P. Bucci 12/C, Arcavacata di Rende (Cosenza) 87036, Italy

**Keywords:** *Arabidopsis thaliana*, carbonylation, isobenzofuranimines, shoot parameters, root morphology, phytotoxicity

## Abstract

A novel class of potential herbicides, the 3-(methoxycarbonylmethylene) isobenzofuran-1-imines, has been discovered. The herbicidal activity has been tested on two particular molecules, (*E*)-methyl 2-[3-(butylimino)isobenzofuran-1(3*H*)-ylidene]acetate (**1**) and (*E*)-methyl 2-phenyl-2-[3-(phenylimino)isobenzofuran-1(3*H*)-ylidene]acetate (**2**), prepared by palladium-catalyzed oxidative carbonylation of 2-alkynylbenzamides. Both compounds **1** and **2** showed a strong phytotoxic effect on both shoot and root systems of *Arabidopsis thaliana*. The effects observed on the shoot were similar for both molecules, but while compound **1** showed a stronger effect on root parameters (such as primary root length, root hair and density, showing lower ED_50_ values), compound **2** caused important malformations in root morphology. Our results indicate that these molecules are very promising synthetic herbicides.

## 1. Introduction

In modern agriculture, the control of weeds is one of the goals to maximize crop yield and quality. Weeds compete with crops not only for edaphic resources such as water and nutrients, but also for space and sunlight. Moreover, many weeds are the hosts of a variety of pathogens such as viruses, fungi, insects, which in turn can determine the occurrence and spread of plant diseases and insect pests in crops. Nowadays, herbicides play a pivotal role in management strategies in weed control and billions of dollars are spent every year to cope with this problem.

Since the 1940s, when the first synthetic chemicals were introduced for this purpose, the agrochemical industry has successfully developed a wide array of herbicides, characterized by various chemical structures and modes of action [1]. However, in recent decades, the indiscriminate use of chemicals has caused the development of herbicide-resistant weeds [2]. Ryan was the first to describe simazine resistance in *Senecio vulgaris* [3]. Later, it has been demonstrated that chlorsulfuron, atrazine, diclofop-methyl, and paraquat caused a significant change in weed communities and consequently the evolution of herbicide-resistant biotypes insensitive to herbicidal treatments [4]. Since 1990, weed-resistance to most herbicide classes in almost 100 weed species has been described [4]. Therefore, the research aimed at developing new synthetic herbicides, with novel modes of action compared to the currently used herbicides, is becoming more and more important. Although this approach is an useful strategy for weed management, especially in recent years in which the number of patents on herbicides has dramatically decreased, repeated application of effective herbicides with the same mode of action continues to be the greatest risk factor for herbicide-resistance evolution [5]. That is why the development of new herbicides with multiple modes of action, or of suitable mixtures of herbicides able to simultaneously affect different targets, is becoming of primary importance in the current weed management research [6].

In this paper, the biological activity of two synthetic 3-(methoxycarbonylmethylene)isobenzofuran-1-imine derivatives, (*E*)-methyl 2-[3-(butylimino)isobenzofuran-1(3*H*)-ylidene]acetate (**1**) and (*E*)-methyl 2-phenyl-2-[3-(phenylimino)isobenzofuran-1(3*H*)-ylidene]acetate (**2**), on plant growth and metabolism of *Arabidopsis thaliana* has been evaluated. These molecules, synthetized from simple substrates by a catalytic carbonylation approach, showed significant and very promising potential herbicidal activity.

## 2. Results and Discussion

### 2.1. Synthesis of 3-(Methoxycarbonylmethylene)isobenzofuran-1-imines **1** and **2**

**Scheme 1:**
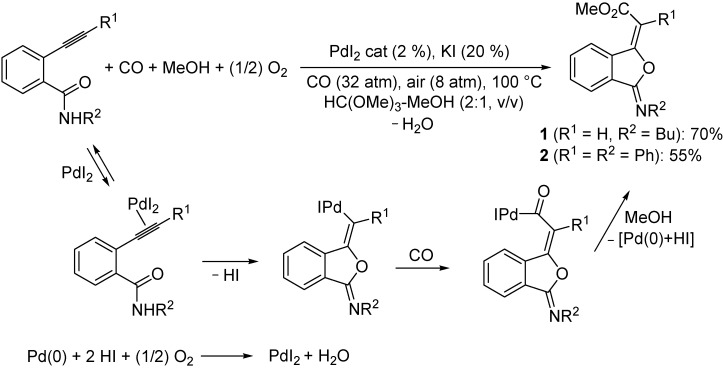
.Synthesis of (*E*)-methyl 2-[3-(butylimino)isobenzofuran-1(3*H*)-ylidene]acetate (**1**) and (*E*)-methyl 2-phenyl-2-[3-(phenylimino)isobenzofuran-1(3*H*)-ylidene]acetate (**2**) by PdI_2_/KI-catalyzed oxidative carbonylation of *N*-butyl-2-ethynylbenzamide and *N*-phenyl-2-(2-phenylethynyl)benzamide, respectively (anionic iodide ligands are omitted for clarity).

(*E*)-Methyl 2-[3-(butylimino)isobenzofuran-1(3*H*)-ylidene]acetate (**1**) and (*E*)-methyl 2-phenyl-2-[3-(phenylimino)isobenzofuran-1(3*H*)-ylidene]acetate (**2**) were prepared by PdI_2_/KI-catalyzed oxidative carbonylation [7] of *N*-butyl-2-ethynylbenzamide and *N*-phenyl-2-(2-phenylethynyl)benzamide, respectively, carried out in a trimethyl orthoformate/MeOH mixture (2:1 v/v) at 100 °C and under 40 atm of a 4:1 mixture of CO-air, in the presence of 2 mol % of PdI_2_ and 20 mol % of KI, according to Scheme 1 (anionic iodide ligands are omitted for clarity). The use of trimethyl orthoformate as co-solvent was necessary in order to avoid substrate hydrolysis under the reaction conditions. The process occurs through *anti* 5-*exo*-*dig* *O*-cyclization, ensuing from intramolecular nucleophilic attack by the carbonyl oxygen on triple bond coordinated to PdI_2_, followed by carbon monoxide insertion and nucleophilic displacement by MeOH (Scheme 1). The Pd(0) species formed in the nucleophilic displacement step is then reoxidized back to PdI_2_ by the action of O_2_ according to a well-known mechanism [7,8,9].

### 2.2. Effects of 3-(Methoxycarbonylmethylene)isobenzofuran-1-imines **1** and **2** on A. thaliana Shoot Morpho-Physiological Parameters

**Figure 1:**
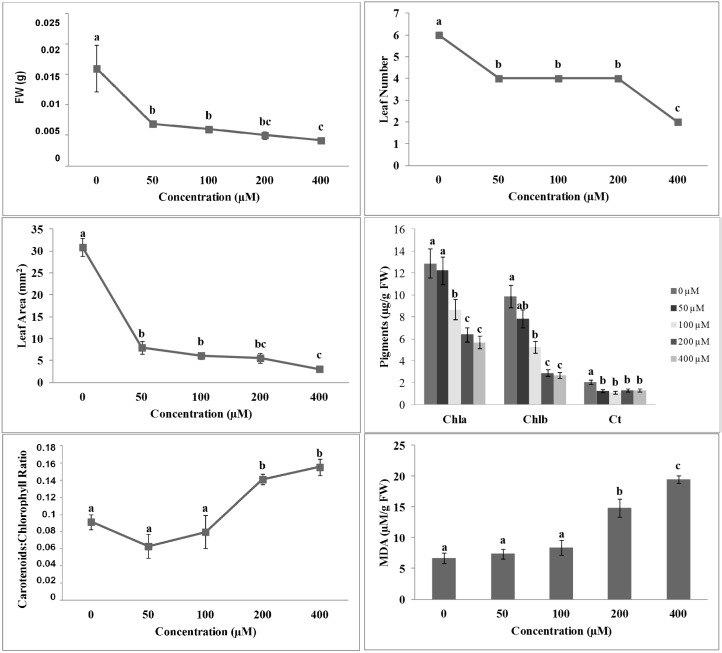
Morpho-physiological parameters of *A. thaliana* seedlings treated with different concentrations of compound **1**: Shoot Fresh Weight (SFW), Leaf Number (LN), Leaf Area (LA), pigments content, Carotenoids:Chlorophyll Ratio and MDA content. Different letters along the curves indicate significant differences at *p* ≤ 0.05. All data were analyzed by ANOVA, applying Tukey’s and Tamhane’s T2 tests for homoscedastic and heteroscedastic parameters, respectively (*N* = 5).

**Figure 2:**
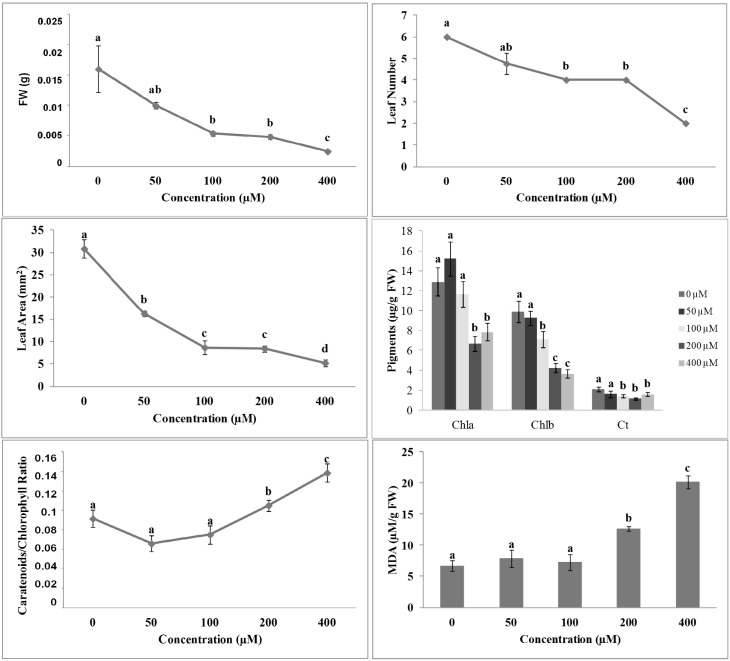
Morpho-physiological parameters of *A. thaliana* seedlings treated with different concentrations of the compound **2**: Shoot Fresh Weight (SFW), Leaf Number (LN), Leaf Area (LA), pigments content, Carotenoids:Chlorophyll Ratio and MDA content. Different letters along the curves indicate significant differences at *p* ≤ 0.05. All data were analyzed by ANOVA, applying Tukey’s and Tamhane’s T2 tests for homoscedastic and heteroscedastic parameters, respectively (*N* = 5).

To verify if compounds **1** and **2** could be active as potential new herbicides, we tested them in a rather broad range of concentrations (50–400 µM), as reported in the literature for other newly synthesized molecules [10]. Both molecules **1** and **2** caused a strong inhibitory effect on all morpho-physiological parameters in *A. thaliana* seedlings. In particular, shoot fresh weight (SFW) was significantly affected by **1** already at the lowest concentration tested (50 µM) (Figure 1), whereas **2** caused a significant inhibition at 100 µM (Figure 2). The complete inhibition was reached at the highest concentration tested (400 µM) with both molecules (Figure 1 and Figure 2).

The non-linear regression fits of the SFW dose-response curves of **1** and **2** were characterized by a high statistical significance (*p* ≤ 0.001), but differed significantly in ED_50_ values (20 µM *vs.* 70 µM, respectively), confirming the higher inhibitory activity of **1** compared to **2** (data not shown). A similar trend of inhibition on Leaf Area (LA) and Leaf Number (LN) parameters was observed (Figure 1 and Figure 2).

The simultaneous decrease in SFW, LA and LN suggested a more complex effect induced by both isobenzofuranimines **1** and **2** on the overall plant metabolism. Indeed, both molecules reduced, with a similar trend, the pigments content (Figure 1 and Figure 2). In particular, chlorophyll *a* and *b* content decreased in a dose-dependent manner, whereas the carotenoid content was decreased at all tested concentrations (Figure 1 and Figure 2). Previous studies already demonstrated a reduction in chlorophyll *a* and *b* contents coupled with a chlorotic appearance in the leaves of plants treated with phenolic acids [11,12].

Recently, similar results were also reported on *Arabidopsis* plants treated with citral [13], a volatile component of many aromatic plants [14]. In particular, the authors observed that citral treatment caused strong inhibitory effects on several photosynthetic parameters, such as the pigment content and fluorescence of the chlorophyll *a*. The carotenoids/chlorophyll ratio significantly increased upon treatment with 200 and 400 µM of both molecules. A similar trend was followed by the malonyldialdehyde (MDA) content (Figure 1 and Figure 2). As reported by Filella *et al.* [15], carotenoids/chlorophyll ratio index increased under abnormal or limiting conditions. The change in ratio index could be due to an increase in carotenoids content, indicating an activation of the photoprotection mechanism by plants. Conversely, it could be due to a reduction in chlorophylls content, meaning the occurrence of photodegradation phenomena.

Under our experimental conditions, the change in carotenoids/chlorophyll ratio index appeared to be caused by a reduction in chlorophyll *a* and *b* content accompanied by an increase in MDA, suggesting that the photodegradation could be attributable to oxidation damages. A similar effect was observed on *Arabidopsis* plants treated with 2-3*H*-benzoxazolinone (BOA) [16]: an increase in lipid peroxidation was accompanied by a proteins and pigments degradation, which has been associated with a reduction in stability of the antenna complex, as also suggested by Liu *et al.* [17].

### 2.3. Effects of Isobenzofuranimines **1** and **2** on Root Morphology of A. thaliana

**Figure 3:**
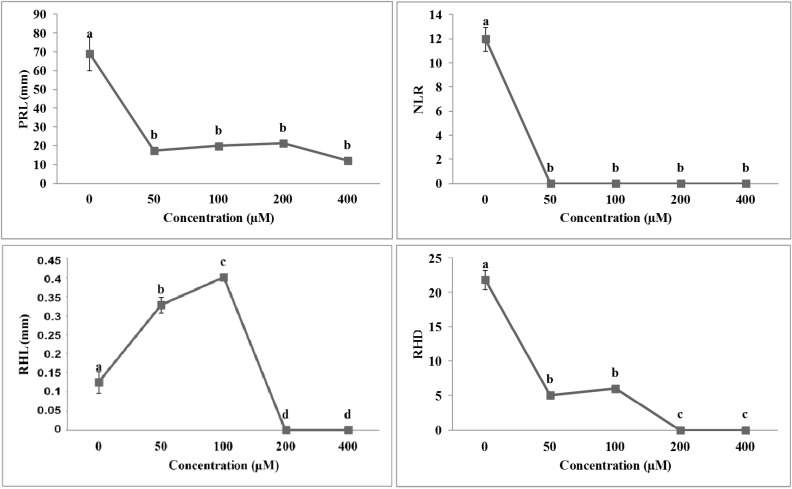
Dose-response curves of root morphology of *A. thaliana* seedlings treated with compound **1**: primary root length (PRL), number of lateral root (NLR), root hair density (RHD) and root hair length (RHL). Data are expressed as percentage of the control. Different letters along the curves indicate significant differences at *p* ≤ 0.05. All data were analyzed by ANOVA, applying Tukey’s and Tamhane’s T2 tests for homoscedastic and heteroscedastic parameters, respectively (*N* = 5).

**Figure 4:**
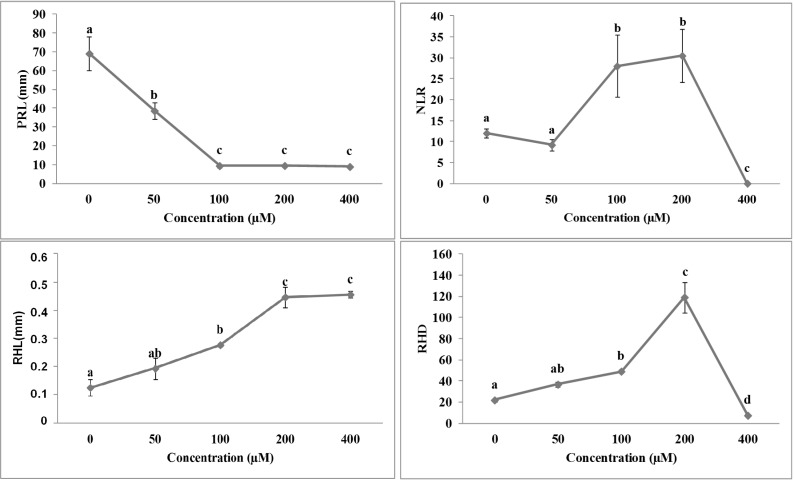
Dose-response curves of root morphology of *A. thaliana* seedlings treated for 10 days with compound **2**: primary root length (PRL), number of lateral root (NLR), root hair density (RHD) and root hair length (RHL). Data are expressed as percentage of the control. Different letters along the curves indicate significant differences at *p* ≤ 0.05). All data were analyzed by ANOVA, applying Tukey’s and Tamhane’s T2 tests for homoscedastic and heteroscedastic parameters, respectively (*N* = 5).

The root morphology of *A. thaliana* seedlings was strongly affected by both isobenzofuranimines **1** and **2** (Figure 3 and Figure 4). Both molecules were effective at the lowest concentration tested, causing a reduction in primary root length by 75% and 45% for molecule **1** and **2**, respectively (Figure 3 and Figure 4).

On the other hand, compounds **1** and **2** had a contrasting effect on NLR. In particular, seedlings treated with **1** showed a lack in lateral roots formation already at the lowest concentration, whereas those treated with **2** showed a significant stimulation (*hormetic* effect) between 100 and 200 µM concentrations range, reaching the complete inhibition only at 400 µM (Figure 3). Interestingly, both the stimulatory and inhibitory effects were also observed on both root hairs parameters (RHD and RHL). In particular, at 50 and 100 µM concentrations, molecule **1** caused a reduction of root hair density (RHD), which was accompanied by an increase in root hair length (RHL), whereas higher concentrations completely inhibited both parameters (Figure 3). On the other hand, molecule **2** significantly stimulated the RHL parameter at all the concentrations tested (Figure 4), whereas the RHD significantly increased up to 200 µM concentration and then collapsed at the highest concentration (Figure 4). The non-linear regression fit of data and the comparison of the ED_50_ values (Table 1) pointed out a higher inhibitory effect of isobenzofuranimine **1** compared to **2**, in all the root parameters, already at lower concentrations (Table 1). Conversely, compound **2** was able to stimulate NLR and RHD parameters at lower concentrations than **1,** which in its turn was able to stimulate RHL.

**Table 1:** ED_50_ (µM) values and stimulatory concentrations (µM) of primary root length (PRL), number of lateral root (NLR), root hair density (RHD) and root hair length (RHL) of *A. thaliana* estimated by the log-logistic equations in response to different concentrations of compounds **1** and **2**. Data from Figure 1, Figure 2, Figure 3 and Figure 4. Different letters along the columns, for each parameter, indicate significant differences at *p* < 0.05 (Tukey’s test). Values within the brackets indicated the standard deviation (*N* = 5). All the dose-response curves pointed out a significance level of *p* < 0.001.

	ED_50_ (µM)			
Isobenzofuranimine	PRL	NLR	RHD	RHL
**1**	28.5 (±0.46) ^a^	9.73 * e-13 (±0.0001) ^a^	20.46 (±1.5) ^a^	119.87 (±11.78) ^a^
**2**	52.5 (±4.8) ^b^	225.06 (±5.29) ^b^	397.1 (±0.78) ^b^	ND
	**Stimulation (µM)**			
**1**	ND	ND	ND	18.16 (±0.21) ^a^
**2**	ND	28.66 (±5.3)	60.97 (±0.04)	108.2 (±2.1) ^b^

**Figure 5:**
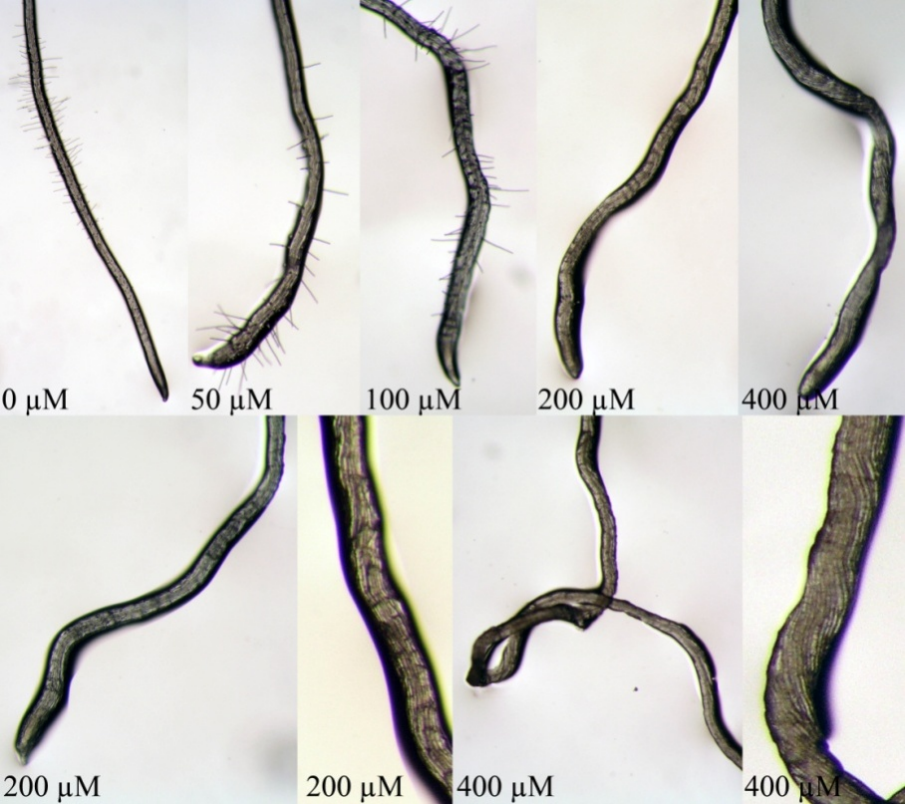
Root tip of *A. thaliana* grown *in vitro* and treated with different concentrations of compound **1**. Note the lack of root hairs development at the highest concentrations. NB photos taken with different magnification.

**Figure 6:**
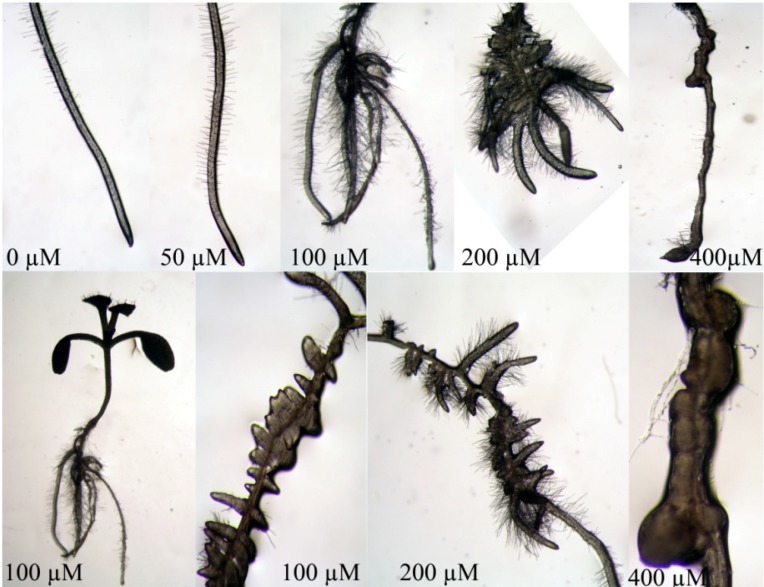
Root tip of *A. thaliana* grown *in vitro* and treated with different concentrations of compound **2**. Note the root malformation and the stimulation of lateral roots and root hairs. NB photos taken with different magnification.

Although similar in chemical structure, the two molecules induced contrasting effects on the root morphology and anatomy (compare Figure 5 and Figure 6). In particular, seedlings treated with isobenzofuranimine **1**, especially at high concentrations (200 and 400 µM), caused a strong reduction in root growth, accompanied by a lack in lateral roots and root hairs. Moreover, root deformations, mainly caused by not aligned cells, resulted evident (Figure 5). These effects are typically induced by okadaic acid and calyculin-A, protein-phosphatase inhibitors, which are able to block the root hair development, and severely affect the shape of the cells within the zone of elongation, inhibit root growth rates and change the root directional growth [18]. These negative modifications in root development are ascribable to the ability of these molecules to inhibit the dephosphorylation of phosphoproteins by members of the type-1 and -2A family of protein phosphatases [19,20], required for the cell-cycle progression and the control of microtubule reorganization during mitosis [21,22,23]. In addition, the lack in root hair development was constantly observed in seedlings treated with microtubules effectors [24,25], confirming the possibility that compound **1**, at the concentrations of 200 and 400 µM, could indirectly interfere with the microtubule stability. Finally, it cannot be excluded that the hormonal imbalance could be responsible of the lack of lateral roots, as suggested by Ivanchenko *et al.* [26].

The morphological and anatomical effects observed upon treatment with isobenzofuranimine **2** are reported in Figure 6. Remarkably, the effects caused by this molecule were opposite with respect to those of compound **1**. Indeed, seedlings treated with 100 and 200 µM concentrations, showed an increase of the lateral roots number, length and density of root hairs, along with strong malformations mainly due to the growing fused or irregularly spaced lateral roots (Figure 6). Moreover, the distribution of lateral roots along the axes appeared to be rather grouped in clusters, losing the normal longitudinal positioning observable in the control (Figure 6).

At the highest concentration (400 µM), a strong swelling of the root tip was evident and, along root axes, an attempted differentiation of lateral roots, whose growth was blocked leading to primary root deformation. Similar results were also observed by Li *et al.* [27], when *Arabidopsis* seedlings were treated with 4-methylumbelliferone. An increased expression of two auxin efflux facilitator genes (PIN2 and PIN3) were observed together with a lack of response of the auxin receptor TIR1 and the key auxin biosynthetic gene YUCCA1. These results suggested that root branching stimulation could be due to a direct or indirect mediation of auxin redistribution, rather than auxin biosynthesis. Moreover, PIN proteins affected lateral root density and spacing. They observed in a triple PIN mutant the reduction of longitudinal lateral roots spacing. Furthermore, the increase in lateral root density was not accompanied by a concurrent increase in root length.

Accordingly to our results, Lakowski *et al.* [28] also observed a high number of fused lateral roots, which were separated into two distinct roots only on the tips, indicating that in triple PIN mutant the mechanisms leading to lateral inhibition of organ formation could be interrupted.

Further studies are needed to understand the mode of action of both molecules. Since an involvement of plant growth regulators have been hypothesized, the use of *Arabidopsis* mutants and/or auxin transport and biosynthesis inhibitors could help to better explain the isobenzofuranimine mode of action.

## 3. Experimental

### 3.1. General Information

Chemicals were purchased from Sigma-Aldrich Italia (Milano, Italy) and were used as such without further purification. Melting points were taken on a Reichert Thermovar apparatus and are uncorrected. ^1^H-NMR and ^13^C-NMR spectra were recorded at 25 °C in CDCl_3_ solutions with a Bruker DPX Avance 300 spectrometer operating at 300 MHz and 75 MHz, respectively, with Me_4_Si as internal standard. Chemical shifts (δ) and coupling constants (*J*) are given in ppm and in Hz, respectively. IR spectra were taken with a JASCO FT-IR 4200 spectrometer. Mass spectra were obtained using a Shimadzu QP-2010 GC-MS apparatus at 70 eV ionization voltage. Microanalyses were carried out with a Carlo Erba Elemental Analyzer Mod. 1106. All reactions were analyzed by TLC on silica gel 60 F_254_ (Merck) or on neutral alumina (Merck) and by GLC using a Shimadzu GC-2010 gas chromatograph and capillary columns with polymethylsilicone + 5% polyphenylsilicone as the stationary phase (HP-5). Column chromatography was performed on neutral alumina 90 (Merck, 70–230 mesh). Evaporation refers to the removal of solvent under reduced pressure.

### 3.2. Preparation of Isobenzofuranimines **1** and **2** [29]

A 250 mL stainless steel autoclave was charged in the presence of air with PdI_2_ (5.0 mg, 1.39 × 10^−2^ mmol), KI (23.0 mg, 1.39 × 10^−1^ mmol) and a solution of *N*-butyl-2-ethynylbenzamide (141 mg, 0.70 mmol) or *N*-phenyl-2-(2-phenylethynyl)benzamide (208 mg, 0.70 mmol) in a MeOH/HC(OMe)_3_ mixture [MeOH: 11.6 mL; HC(OMe)_3_: 23.1 mL]. The autoclave was sealed and, while the mixture was stirred, the autoclave was pressurized with CO (32 atm) and air (up to 40 atm). After being stirred at 100 °C for the 15 h, the autoclave was cooled, degassed and opened. The solvent was evaporated, and the residue purified by column chromatography on neutral alumina (eluent: 95:5 hexane/AcOEt) to give pure (*E*)-methyl 2-[3-(butylimino)isobenzofuran-1(3*H*)-ylidene]acetate **1** (127.3 mg, 70%) or (*E*)-methyl 2-phenyl-2-[3-(phenylimino)isobenzofuran-1(3*H*)-ylidene]acetate **2** (136.8 mg, 55%).

*(E)-methyl 2-[3-(butylimino)isobenzofuran-1(3H)-ylidene]acetate* (**1**). Colorless solid, mp 106-107 °C. IR (KBr ): *ν* = 2957 (m), 2932 (m), 2873 (w), 1687 (m), 1646 (s), 1582 (s), 1459 (m), 1402 (m), 1316 (w), 1221 (m), 1142 (m), 768 (m) cm^−1^; ^1^H-NMR (300 MHz, CDCl_3_): *δ* = 9.09-9.00 (m, 1 H), 7.92-7.84 (m, 1 H), 7.68-7.56 (m, 2 H), 5.94 (s, 1 H), 3.80 (s, 3 H), 3.66 (t, *J* = 7.2, 2 H), 1.78-1.61 (m, 2 H), 1.52-1.37 (m, 2 H), 0.97 (t, *J* = 7.3, 3 H); ^13^C-NMR (75 MHz, CDCl_3_): *δ* = 167.0, 161.8, 152.8, 133.7, 132.6, 132.1, 132.0, 127.7, 122.6, 96.6, 51.5, 47.9, 32.9, 20.6, 13.9; GC-MS: *m/z* = 259 (35) [M^+^], 228 (13), 200 (100), 186 (29), 184 (26), 172 (29), 159 (44), 158 (83), 130 (41), 102 (16), 89 (18); anal. calcd for C_15_H_17_NO_3_ (259.30): C, 69.48; H, 6.61; N, 5.40; found C, 69.50; H, 6.58; N, 5.39.

*(E)-methyl 2-phenyl-2-[3-(phenylimino)isobenzofuran-1(3H)-ylidene]acetate* **(2**). Yellow solid, mp 56-57 °C; IR (KBr): *ν* = 3019 (w), 1712 (s), 1691(s), 1613 (m), 1590 (m), 1489 (w), 1300 (w), 1268 (w), 1216 (m), 1053 (s), 1006 (m), 757 (s), 692 (w) cm^−1^; ^1^H-NMR (300 MHz, CDCl_3_): *δ* = 8.29 (d, *J* = 7.1, 1 H), 8.07-8.02 (m, 1 H), 7.68-7.57 (m, 2 H), 7.50-7.43 (m, 2 H), 7.43-7.28 (m, 5 H), 7.26-7.19 (m, 2 H), 7.15-7.07 (m, 1 H), 3.89 (s, 3 H); ^13^C-NMR (75 MHz, CDCl_3_): *δ* = 167.8, 153.3, 151.8, 144.5, 134.1, 134.0, 132.4, 132.2, 131.2, 129.5, 128.6, 128.2, 127.9, 125.5, 125.2, 124.9, 123.7, 112.7, 52.5; GC-MS: *m/z* = 355 (36) [M^+^], 324 (16), 296 (100), 295 (46), 267 (21), 246 (5), 219 (5), 190 (7), 165 (7), 77 (16); anal. calcd for C_23_H_17_NO_3_ (355.39): C, 77.73; H, 4.82; N, 3.94; found C, 77.71; H, 4.85; N, 3.90.

### 3.3. Seedlings Growth Bioassay

*Arabidopsis thaliana* L. (Heyn.) seeds, ecotype Columbia (Col-0), were surface sterilized for 3 min in EtOH/Triton X-100 (50:0.01), and successively in NaOCl/Triton X-100 (0.5:0.01) solutions for 3 min, then were rinsed three times in sterilized distilled water. To promote the synchronization of the germination, the seeds were vernalized in 0.1% agar solution at 4 °C for 48 h, and then fifty seeds were sown in Petri dishes (100 mm × 150 mm) containing agar (0.8%) enriched with Murashige–Skoog medium (from Sigma–Aldrich) and sucrose (1%). The Petri dishes were then kept in a growth chamber with 8/16 h [light (60 μmol m^−2^ s^−1^)/darkness] photoperiod, at 22 ± 2 °C and 55% relative humidity for 4 days. To evaluate the effect of each compound on plant growth, six *Arabidopsis* seedlings (4 days old) were transplanted on Petri dish containing 150 mL of agarised medium (0.8%) enriched with 0, 50, 100, 200, 400 μM of each compound. Both molecules were firstly dissolved in EtOH and then diluted in agarized medium to reach the final concentrations. In Control treatment was added the same amount of ethanol employed to solubilize the molecules.

The seedlings were incubated as previously described for 10 days, and then plant material was sampled for the analyses. Total fresh weight (FW), leaf number (LN), total leaf area (LA) and root morphology were analyzed. In particular, Primary Root Length (PRL), Number of Lateral Root (NLR), Root Hair Length (RHL), and Root Hair Density (RHD) were analyzed through the WinRhizo Pro System v. 2002a software (Instruments Règent Inc., Sainte-Foy-Sillery-Cap-Rouge, Quebec, Canada), after capturing roots image by scanner (Epson Expression 800, Instruments Règent).

### 3.4. Measurement of Photosynthetic Pigments

The total amounts of chlorophyll a, chlorophyll b, and carotenoids were analyzed and calculated according to Wellburn [30]. The pigment content (mg/g of FW) was evaluated according to the following Equations [30]:

Chl_a_ (µg) = (15.65 (DO_666_ − DO_750_) − 7.34 (DO_653_ − DO_750_)) * V (1)

Chl_b_ (µg) = (27.05 (DO_653_ − DO_750_) − 11.21 (DO_666_ − DO_750_)) * V (2)

Ct (X+C) (µg) = (1000 (DO_470_ − DO_750_) − 2.86 Chl_a_ − 129.2 Chl_b_)/221) * V (3)

In addition, the carotenoid:chlorophyll ratio was calculated as an indicative measurement of the physiological status of the plants [15].

### 3.5. Lipid Peroxidation

The extent of lipid peroxidation was determined on treated seedlings of *A. thaliana* through measuring the amount of malonyldialdehyde (MDA) formation by thiobarbituric acid method as described by Hodges *et al.* [31]. After treatment, 100 mg of plant material were homogenized in 80% ethanol and centrifuged at 3,000 *×g* for 10 min at 4 °C, the supernatant was collected, incubated for 25 min at 95 °C with 20% trichloroacetic acid (TCA) containing 0.01% hydroxytoluenebutylate, with and without 0.5% thiobarbituric acid (TBA), and quickly cooled in ice for 10 min. The absorbance of the reaction mixture was measured at 450, 532 and 600 nm. The equivalents of MDA (nmol/mL) were calculated based on the following formulae:

A = [(Abs_532+TBA_ − Abs_600+TBA_) − (Abs_532-TBA_ − Abs_600-TBA_)] (4)

B = [(Abs_440+TBA_ − Abs_600+TBA_) * 0.0571] (5)

MDA equivalents (nmol/mL) = (A − B/157000) * 10^6^ (6)

### 3.6. Statistical Analysis

To evaluate the effects of each molecule a completely random design with five replications was adopted. Data were evaluated for normality (Kolmogorov-Smirnov test) and tested for homogeneity of variances (Levene’s test). All parameters were estimated by ANOVA, and Tukey’s (for homoscedastic data) or Tamhane’s T2 (for heteroscedastic data) test (*p* ≤ 0.05) was applied to compare the statistical significance of differences among group means. All statistical analyses were performed using SPSS *ver*. 6.1 software (Insightful Corporation, Seattle, WA, USA). The responses of FW, PRL, NLR, RHL and RHD parameters to different doses of both molecules were evaluated by a nonlinear regression model using the Equation (7) for inhibitory effects, or the Equation (8) when stimulatory (hormesis) effects were observed [32,33]:

*y* = C + {D − C/1 + *e*^[*B* ln(x/ED_50_)]} (7)

*y* = C + {D − C + f * x/1 + [1 + (2 * f * ED50/D − C)] * *e*^[*B* ln(x/ED_50_)]} (8)

where C was the expected response at indefinitely large concentrations, D the control mean response, *f* the initial rate of increase in response at sub-inhibitory concentration, finally ED_50_, defined the dose required to reduce 50% of the total response, where B was the rate of change around the ED_50_.

The ED_50_ data were firstly checked for normality (Kolmogorov-Smirnov test) and tested for homogeneity (Leven Median test). Then, the phytotoxicity comparison between two molecules was performed by one-way ANOVA using the ED_50_ as a variable and the molecule as main factor. The statistical significance of differences among group means were estimated by Tukey’s (homoscedastic data) or Tamhane’s T2 (heteroscedastic data) tests (*p* ≤ 0.05).

## 4. Conclusions

Taken together, our results indicate that 3-(methoxycarbonylmethylene)isobenzofuran-1-imines are a promising potential class of novel synthetic herbicides. In particular, (*E*)-methyl 2-[3- (butylimino)isobenzofuran-1(3*H*)-ylidene]acetate (**1**) and (*E*)-methyl 2-phenyl-2-[3-(phenylimino)-isobenzofuran-1(3*H*)-ylidene]acetate (**2**), synthetized by a catalytic oxidative carbonylation process, are able to induce alterations on plant metabolism at multiple target sites. Both compounds **1** and **2** showed a strong phytotoxic effect on the shoot as well as the root systems of *Arabidopsis* seedlings. The two molecules had different effects on root growth, even though a hormonal involvement has been suggested in both cases. It is possible that the effects on the aerial part, similar for both molecules, are due to an indirect effect of the strong alterations on root morphology and anatomy. It is well known that the root system plays a crucial role on nutrient uptake, and an alteration of its function could be responsible for the limited nutrient supply and consequently for shoot form and function.
